# Aboriginal and Torres Strait Islander Worldviews and Cultural Safety Transforming Sexual Assault Service Provision for Children and Young People

**DOI:** 10.3390/ijerph10093818

**Published:** 2013-08-22

**Authors:** Leticia Funston

**Affiliations:** NSW Health Education Centre Against Violence, Locked Bag 7118, Parramatta BC, NSW 2150, Australia; E-Mail: Leticia.Funston@swahs.health.nsw.gov.au; Tel.: +61-02-9840-3735; Fax: +61-02-9840-3754

**Keywords:** Aboriginal and Torres Strait Islander people, children and young people, child sexual assault, those who sexually harm others, trauma, gender, cultural safety, aboriginal worldviews, aboriginal community-based participation research methods

## Abstract

Child Sexual Assault (CSA) in Aboriginal and Torres Strait Islander communities is a complex issue that cannot be understood in isolation from the ongoing impacts of colonial invasion, genocide, assimilation, institutionalised racism and severe socio-economic deprivation. Service responses to CSA are often experienced as racist, culturally, financially and/or geographically inaccessible. A two-day forum, *National Yarn Up: Sharing the Wisdoms and Challenges of Young People and Sexual Abuse*, was convened by sexual assault services to identify the main practice and policy concerns regarding working with Aboriginal and Torres Strait Islander children and young people (C&YP), families and communities in the context of CSA. The forum also aimed to explore how services can become more accountable and better engaged with the communities they are designed to support. The forum was attended by eighty invited Aboriginal and Torres Strait Islander and non-Aboriginal youth sexual assault managers and workers representing both “victim” and “those who sexually harm others” services. In keeping with Aboriginal Community-Based Research methods forum participants largely directed discussions and contributed to the analysis of key themes and recommendations reported in this article. The need for sexual assault services to prioritise cultural safety by meaningfully integrating Aboriginal and Torres Strait Islander Worldviews emerged as a key recommendation. It was also identified that collaboration between “victims” and “those who sexually harm” services are essential given Aboriginal and Torres Strait Islander C&YP who sexually harm others may have also been victims of sexual assault or physical violence and intergenerational trauma. By working with the whole family and community, a collaborative approach is more likely than the current service model to develop cultural safety and thus increase the accessibility of sexual assault services.

## 1. Introduction

Aboriginal and Torres Strait Islander peoples of Australia represent the oldest surviving cultures in the world [1]. Over thousands of years Aboriginal and Torres Strait Islander societies developed complex and diverse systems of governance, law, health and healing practices spanning hundreds of different countries within the continent [1,2]. While it is not possible to provide a detailed history of Indigenous Australia here, it is important to recognise that prior to British colonial invasion, Aboriginal and Torres Strait Islander children and families were healthy and prosperous [3]. At the time of the invasion in 1770, Aboriginal and Torres Strait Islander people were considered to be much healthier than many of the British people who arrived suffering a raft of afflictions including malnutrition, infectious diseases, alcoholism and violence [3,4,5]. The doctrine of Terra Nullius, a Latin phrase meaning “a land belonging to no one”, came to be used to justify the genocide of Aboriginal and Torres Strait Islander peoples, the forced acquisition of their land and violent attempts to assimilate them into an imposed system and culture [4,5]. These traumatic legacies together with ongoing neo- colonial policies continue to impact Aboriginal and Torres Strait Islander people and communities. Despite the terror and violence perpetrated by the early colonisers and successive Australian governments over the past 225 years, Aboriginal and Torres Strait Islander people continue to have a proud history of resistance, survival and strong culture [4].

According to Judith Herman, violence is “always embedded in a social structure that permits the abuse and exploitation of a subordinate group” ([6], p. xiv). Child sexual assault (CSA) in Aboriginal and Torres Strait Islander families and communities is no exception. The failure to understand the historical, socio-political context of sexual violence has fuelled racist attitudes and policies and has falsely ascribed blame to Aboriginal culture [7]. This failure to understand the complex interaction between sexual violence and the ongoing impacts of colonialism and racism has also been replicated at the level of human services, arguably contributing to the “isolation of communities, protection of abusers, and under-use of mainstream services by Aboriginal people” ([7], p. 1). The recent NSW Ombudsman report on the Interagency Plan to Tackle Child Sexual Assault in Aboriginal Communities reveals that sexual assault services funded to provide support to Aboriginal and Torres Strait Islander C&YP often lack accountability and transparency measures and are insufficiently resourced to respond to CSA particularly in rural and remote areas [8]. Sexual assault services are hampered by ongoing staffing shortages and are generally poorly integrated into the communities they are designed to support [8]. The report also strongly criticises the Interagency Plan for failing to support community capacity building initiatives and for failing to provide a consistent strategy informed by meaningful community consultation [8,9].

Creating accessible and culturally safe sexual assault services for Aboriginal and Torres Strait Islander children and young people (C&YP) as well as changing the social conditions that enable sexual violence to occur, has been a long-standing challenge. The *National Yarn Up: Sharing the Wisdoms and Challenges of Young People and Sexual Abuse* forum, convened by NSW Health Education Centre Against Violence (ECAV; state-wide specialised training, consultancy and resource development for NSW Health and interagency workers who provide services to children and adults who have experienced sexual assault, domestic or Aboriginal family violence and/or physical and emotional abuse and neglect.), Newstreet Services (specialist sexual assault service for children and young people who have sexually harmed others) and the Adolescent Roundtable (Australian and New Zealand forum for service managers working with young people who have sexually harmed others), brought together a range of specialist sexual assault services from various locations across Australia to discuss these practice and policy challenges. This paper reports on the *Yarn Up* participant responses to the following discussion questions, which were posed at the forum:

(1) What are the main issues affecting Aboriginal and Torres Strait Islander C&YP, families and communities in the context of sexual assault?
(2) What approaches could be adopted at a practice level, service level and at a policy level to address the challenges identified and how can services become more accountable and better engaged with the communities they serve?

### 1.1. The Impacts and Prevalence of CSA

Overall rates of CSA in Australia are high; over 5,430 reports of CSA were substantiated across Australia between 2010 and 2011 [10]. However, as many assaults are not disclosed, a much higher prevalence rate is estimated [8,11]. A recent meta-analysis of 55 international studies, reported that CSA prevalence ranges from 8% to 31% for girls and 3% to 17% for boys [9]. This is consistent with the estimated prevalence rate in Australia [12]. However, Aboriginal and Torres Strait C&YP are 6.6 times more likely to be victims of a sexual assault than non-Aboriginal children [8,13] despite comprising a minority of the total population of children [9,14]. There are approximately 294,000 children and young people who identify as Aboriginal and Torres Strait Islander in Australia comprising just 4.2% of the total population of people aged 24 years and younger [15].

The impacts of sexual abuse can be seen in the over representation of Aboriginal and Torres Strait Islander C&YP people in self-harm and suicide statistics [8]. A recent report compiled by the Northern Territory Select Committee on Youth Suicides reveals that the self-harm and suicide rate for Aboriginal and Torres Strait Islander youth is now amongst the highest in the world [16]. Aboriginal and Torres Strait Islander C&YP are also dramatically over-represented in child protection and out of home care populations [17]. According to Elder Rev. Dr. Djiniyini Gondarra, Aboriginal children are being taken away by child protection services at a rate not seen since the Stolen Generations [18]. The “Stolen Generations” refers to the period between 1909 to 1969 where it was official Australian government policy to forcibly remove Aboriginal and Torres Strait Islander children from their families as a strategy to assimilate them into the White Australian culture [19].

The rate of C&YP who sexually harm other children in Australia is also high. The term “those who sexually harm others” is viewed as a more appropriate term for C&YP as it names the harmful behaviour while avoiding totalising identity labels such as “perpetrator” or “offender” [20]. In general, sibling sexual abuse, mostly committed by boys and young men, is more prevalent than CSA perpetrated by step fathers and fathers [21]. In NSW, Aboriginal C&YP who have sexually harmed their siblings comprises 3.9% of all reported juvenile sexual assaults [8]. It is important to emphasise that while sexually harming behaviours is a strong indicator of CSA, not all C&YP who have sexually harmed are also victims. However it is very likely that many Aboriginal and Torres Strait Islander C&YP who sexually harm others have also been victims, if not of sexual and physical violence, then of intergenerational trauma and systemic racism [8]. Young people who sexually harm others are also extremely vulnerable to (re)victimisation as well as re-harming others especially while in Out of Home Care and Juvenile Justice [21]. This is especially concerning given that Aboriginal and Torres Strait Islander young people are vastly over-represented in the Juvenile Justice system ‒ approximately 40% of young people in detention identify as Aboriginal [22]. Aboriginal and Torres Strait Islander young people are also 14 times more likely to be under a community-based supervision order than non-Aboriginal young people [23,24].

### 1.2. The National Yarn up Forum

The national *Yarn Up* forum was organised following the annual Adolescent Roundtable [endorsed by the Australian and New Zealand Association for the Treatment of Sexual Abuse (ANZATSA)] forum held in 2011 for service providers working with children who sexually harm others. The organisers of the *Yarn Up* aimed to expand the focus of Adolescent Roundtable to include children and young people who sexually harm others in Aboriginal and Torres Strait Islander communities. The forum organisers saw the *Yarn Up* as an opportunity to invite sexual assault services working with C&YP victims and those who sexually harm others in order to facilitate greater communication and coordination between these traditionally isolated sectors. Although there are hundreds of sexual assault services in Australia, the forum organisers only invited representatives from sexual assault services that demonstrated an active commitment to engaging Aboriginal and Torres Strait Islander clients and building a strong Aboriginal and Torres Strait Islander work force. The forum organisers also encouraged and prioritised the participation of Aboriginal and Torres Strait Islander sexual assault workers.

The *Yarn Up* forum was held over two days in 2012 at the Mercure Hotel in Sydney and included eighty representatives from services for victims and those who sexually harm others. The *Yarn Up* was also inclusive of Aboriginal and Torres Strait Islander men and women, as well as non-Aboriginal workers recognising the significance of alliances across cultures and genders to this dialogue. Overall, 75% of the forum participants identified as female and 25% identified as male. Half of the female participants and one third of the male participants identified as Aboriginal and or Torres Strait Islander. Although more women attended the forum than men, this may reflect the boader Australian sexual assault service sector, which generally employs a higher proportion of women. However the organisers from ECAV and Newstreet Services argue that there is increasing interest from Aboriginal and Torres Strait Islander men to become engaged in challenging CSA and other forms of violence in Aboriginal communities.

The forum organisers aimed to prioritise the cultural safety of participants by incorporating Aboriginal and Torres Strait Islander Worldviews and customs into the forum structure. The term “Aboriginal and Torres Strait Islander Worldviews” refers to the diverse systems of belief reflecting the hundreds of cultures and laws “that have been sung into a (spiritual) relationship and attachment with the land” ([2], p. 37). Aboriginal and Torres Strait Islander facilitators led all forum discussions. For example, respected Elder and Educational Consultant, Melva Kennedy, opened the *Yarn Up* forum with a Welcome to Country. Mareese Terare, academic and Consultant for Cultural Competency and Healing, presented on the Aboriginal concept of “Dadirri”, which may be understood as the practice of “deep and respectful listening” [25]. Rowena Lawrie, an Aboriginal Clinical Consultant presented on the prevalence of CSA and the socio-political context of violence in Aboriginal and Torres Strait Islander families and communities. The forum was also organised around two “yarning circles” sessions based on Aboriginal and Torres Strait Islander cultural practice. Yarning can be understood as a respectful and informal conversation where knowledge is shared, often through story telling [26]. Pam Greer, a respected Aboriginal Elder and Educational Consultant and Ivan Clarke, Senior Aboriginal Counsellor facilitated group activities based on Aboriginal healing to close each day of the forum. Pam Greer handed each forum participant a dried gum tree leaf from Taree, a township on the NSW mid-north coast widely affected by family violence and CSA, and asked participants to burn the leaf as an act of remembrance and mourning. These Aboriginal customs helped to provide a supportive, culturally safe and equitable space for participants to debrief and reflect on the forum discussions. The strong focus on Aboriginal and Torres Strait Islander worldview and cultural safety also created a space for non-Aboriginal participants to respectfully listen and learn from the knowledge and expertise of the Aboriginal and Torres Strait Islander participants.

## 2. Experimental Section

The main aim of the *Yarn Up* forum was to bring together sexual assault service providers working with Aboriginal and Torres Strait Islander C&YP to discuss key practice and policy challenges. However the organisers of the forum felt that the forum discussions should be documented and disseminated via a publication to extend the conversation to a broader audience. While no formal ethics approval was sought for this project the forum organisers in collaboration with the ECAV researcher adhered to the principles outlined in the *National Statement on Ethical Conduct in Research Involving Humans* [27] and the *Guidelines for Ethical Conduct in Aboriginal and Torres Strait Islander Health Research* [28]. Specifically, the *Yarn Up* forum was informed by the belief in the “right of Indigenous peoples to define, conceptualise, and assess (social problems) within their own cultural systems” ([29], p. 43). The forum drew from the principle that any research involving Aboriginal and Torres Strait Islander people must be directly beneficial to the Aboriginal and Torres Strait Islander participants involved and “committed to dialogue and community self-determination” ([29], p. 44). In light of these understandings, the *Yarn Up* utilised an Aboriginal Community Based Participatory Research method [30]. Aboriginal Community Based Participatory Research is a modified focus group where an “Aboriginal collective voice” determines the direction of the discussion as opposed to the discussion being determined by the researcher [29]. The *Yarn Up* forum structure therefore encouraged participants to actively determine the direction of discussions and also encouraged divergent views [29,30].

The forum organisers chose to record the forum discussions with the aid of a researcher from the ECAV who took detailed notes. The forum discussions were recorded with note taking as it was felt that the presence of recording equipment might make some participants feel uncomfortable. At the beginning of the forum participants were informed that the discussion would be recorded and the notes from the discussions would be used to produce a discussion paper. Participants were also informed that their names would not be disclosed and they had the right to ask the researcher to refrain from documenting any aspects of the discussion if they felt this necessary. All participants consented to this process.

The notes taken during the forum were analysed with NVivo 10 software. Through this process the researcher identified several key themes and created categories. In order to enhance the reliability of the research findings, all forum participants were invited to comment on the key themes identified by the researcher. The preliminary findings were presented to members of the Aboriginal Communities Matter Advisory Group (ACMAG). During this presentation and discussion the ACMAG participants decided on the key themes reported in this article. This method aimed to empower participants through increased ownership over the research process, analysis and distribution of findings.

## 3. Results and Discussion

### 3.1. Invisible Trauma: “We Call that Lack of Justice Not Lack of Trust”

Many of the Aboriginal participants described the trauma experienced from the widespread denial of the history of invasion, genocide and ongoing oppression of Aboriginal and Torres Strait Islander people in Australia. One Aboriginal worker said that while her Jewish friend’s experience of intergenerational trauma related to her parents’ experience of the Shoah (Holocaust) is recognised by the general community, the trauma experienced by Aboriginal and Torres Strait Islander people is never validated or acknowledged. Participants argued that this unacknowledged or “invisible trauma” needs to be better recognised by service providers.

While many non-Aboriginal workers identified themselves as allies committed to anti-racism and equity, participants recognised the importance of formal compulsory education and training to ensure that all sexual assault workers have a strong understanding of how the convergence of historical and contemporary injustices shapes the lives of Aboriginal and Torres Strait Islander people. Participants suggested the following topics should be included in the training; the history of genocide committed against Aboriginal and Torres Strait Islander people, the impacts of racism experienced within the workplace, white privilege, systemic racism, and acculturation stress.

Forum participants discussed at length the immense barriers that Aboriginal and Torres Strait Islander C&YP experience while attempting to disclose sexual assault. Most participants agreed that the disclosure process is greatly impeded by the complex intersection of CSA trauma and relentless social injustices. One participant stated that Aboriginal and Torres Strait Islander people face overwhelming difficulties engaging with and making disclosures to mainstream services in communities where massacres have occurred. Participants suggested that services should assume that Aboriginal and Torres Strait Islander families and communities are going to be reserved about engaging. Services need to demonstrate a genuine commitment to establishing some trust ‒ this may mean leaving the confines of the service and meeting in places Aboriginal C&YP and their families feel comfortable.

Trauma stemming from the Stolen Generations and the current high removal rate of Aboriginal children from their families has contributed to intense fears about the statutory child protection system. Participants noted that Aboriginal families regularly convey intense fears that their children will be removed and that their children may be further abused, experience racism and acculturation stress in institutional care or Out-Of-Home-Care settings.

Participants described how Aboriginal and Torres Strait Islander C&YP often feel unable to disclose abuse when a family, community member or Elder is the perpetrator of the abuse. The mass incarceration of Aboriginal and Torres Strait Islander men and women, excessive police surveillance, together with deaths in custody compels many families and community members to stifle disclosures made by C&YP through threat of “payback” (an aspect of Aboriginal Customary Law, which may be understood as intimidation tactics, including verbal and physical threats). One Aboriginal worker stated “Aboriginal women will protect our men no matter what… we don’t want to subject our families to the Western world… to be killed and hurt in prisons”.

Participants observed how pervasive racist attitudes also create barriers for children to disclose. One participant stated that when an Aboriginal child has been brave enough to disclose sexual assault, the Australian Government and mainstream media frequently labels their family and community as “dysfunctional, diseased and disadvantaged”. Participants recommended that services acknowledge and convey respect to C&YP and their supportive families for the enormous personal strength and courage in making disclosures. They recommended that a strengths-based approach must always be used in working with young people, families and communities.

### 3.2. The Need to Create Cultural Safety by Integrating Aboriginal Worldviews into Mainstream Service Provision

Participants felt that the Western theories currently governing the child protection system are both “inappropriate and colonial”. Aboriginal participants emphasised that in order to decolonise services and create cultural safety within the service system, it is necessary to view violence as a product of the socio-political context. Participants felt that this perspective could dismantle the racist and ahistorical belief that violence is a product of Aboriginal and Torres Strait Islander cultures.

Participants stated that in general, services demonstrate little or no understanding of Aboriginal and Torres Strait Islander cultures and Worldviews. This is evident in the failure of many services to develop protocols with Aboriginal communities for working with C&YP. Participants recommended that any services working with Aboriginal and Torres Strait Islander communities, spend a minimum of twelve months yarning with the community to learn about their culture, history and goals, and to find out what can be done to meet their needs.

Some participants discussed the need for services to engage Aboriginal and Torres Strait Islander Elders in order to demonstrate cultural safety. However many participants reflected that services must firstly develop an understanding of the complex power dynamics operating within communities. Participants suggested that uncritically recommending that services engage with all Elders is overly simplistic. For instance, some Elders may also be perpetrators of CSA and there may be existing conflicts between families and community groups. In addition participants expressed that many Elders do not have any “real” authority within communities to challenge violence and there is currently no support provided to Elders or community leaders from services.

Taking steps towards meaningful engagement with Aboriginal and Torres Strait Islander communities was perceived to be much more effective than “staying within the professional networks”. Yarning with Aboriginal and Torres Strait Islander people in informal community spaces outside of formal service settings was also recommended. It was understood that this form of communication would be more valuable than demonstrating “professional expertise”.

Participants recommended that “mainstream” services meaningfully incorporate Aboriginal and Torres Strait Islander Worldviews into service delivery. Aboriginal participants described their worldviews as “central” to developing cultural safety. One Aboriginal participant expressed this by stating “we need white people listening and to take on Aboriginal principles, then it would be safe to do the healing”. Participants recommended that services should aim to foster pride in Aboriginal and Torres Strait Islander cultures, emphasising stories of healing, resilience and resistance, protest and survival.

### 3.3. Cultural Safety and Supporting Aboriginal and Torres Strait Islander Sexual Assault Workers

Participants discussed the multiple tensions and complexities that may be experienced by Aboriginal and Torres Strait Islander sexual assault workers associated with living in the communities they are working in. Participants described Aboriginal and Torres Strait Islander workers enduring the stress of “living in two worlds”, navigating Aboriginal and Torres Strait Islander Worldviews alongside the Western system. Participants suggested that many Aboriginal and Torres Strait Islander workers who have fair skin experience lateral violence and discrimination from other Aboriginal and non- Aboriginal communities who may question their Aboriginal identity. Lateral violence is the name given to the harmful and undermining practices that “members of oppressed groups can perpetrate against each other as a result of marginalization…colonisation and continued dispossession” ([31], p. 13). Lateral violence, particularly about Aboriginal identity, causes conflicts between Aboriginal and Torres Strait Islander workers and fractures between Aboriginal workers and Aboriginal communities [31]. As one Aboriginal worker reflected, “we haven’t really considered the implications of the stolen generations on our own people”.

Some participants suggested that due to fears around confidentiality Aboriginal and Torres Strait Islander C&YP may feel uncomfortable working with an Aboriginal worker or interpreter who they may be related to or otherwise know. However, other forum participants argued that this issue could be addressed by increasing the Aboriginal and Torres Strait Islander sexual assault work force so C&YP have greater choice and control regarding whom they work with. Participants also identified that it is a significant issue that many Aboriginal and Torres Strait Islander C&YP and their families want to access Aboriginal and Torres Strait Islander workers but cannot because the service has not employed Aboriginal staff members. Participants recommended that training and employing more Aboriginal and Torres Strait Islander specialist sexual assault workers and interpreters is essential. Furthermore, it was recommended that all Aboriginal and Torres Strait Islander staff receive ongoing cultural supervision and support.

### 3.4. Cultural Safety and Supporting Non-Aboriginal Sexual Assault Workers

Participants described a number of challenges experienced by non-Aboriginal workers. For instance one worker said that they have witnessed many non-Aboriginal workers “close down” and disengage from Aboriginal and Torres Strait Islander people and communities out of fear of being perceived as racist. Another participant suggested that “white, new graduate teachers and sexual assault workers” who travel from urban to rural and remote areas for work are often extremely overwhelmed by the poverty, the extent of gender-based violence and CSA in Aboriginal and Torres Strait Islander communities. Participants said that they have seen many non-Aboriginal workers, in an anxious attempt to appear “non-racist”, act especially “nice” to their Aboriginal and Torres Strait Islander colleagues and clients. One participant argued that this excessively “nice” behaviour is often experienced as patronising by Aboriginal and Torres Strait Islander people.

Many non-Aboriginal participants said that they were very interested in learning about Aboriginal and Torres Strait Islander Worldviews. They acknowledged that they currently lack understanding of these beliefs and how they could be integrated into practice. In order to address these gaps in understanding, participants strongly recommended that all non-Aboriginal sexual assault workers receive compulsory training, education and ongoing cultural safety supervision.

### 3.5. Gender Considerations and Responding to Aboriginal and Torres Strait Islander Victims of Sexual Assault and Those Who Sexually Harm

Participants said that Aboriginal and Torres Strait Islander C&YP often experience “double trauma”, where the victim experiences a secondary trauma from the stigmatizing social framing of sexual assault. This secondary assault was described by participants as oppressive as it denies the criminality of child abuse by implying that children and young people are somehow complicit and responsible for their sexual abuse. For example, Aboriginal and Torres Strait Islander male victims of sexual assault are often subjected to ostracism, physical violence and homophobic taunts. For many Aboriginal and Torres Strait Islander young men their assault is not recognised as a crime rather it is constructed as “gay sex”. Several participants described situations where boys under the age of ten who have been sexually abused are labelled “man-made poofter-boys”. Participants also noted that Aboriginal and Torres Strait Islander girls and young women are similarly blamed for their abuse and labelled “sluts”. Participants recommended that community development and education approaches would be useful in countering some of these beliefs. Participants also suggested sexual assault resources and programs need to be gender-specific to reflect the gendered experience of both CSA and double trauma.

A senior Aboriginal educator said that while working with young Aboriginal and Torres Strait Islander men in Juvenile Justice, almost all openly aspired to being incarcerated in “the big house” (prison). A senior Aboriginal worker said that in her experience young Aboriginal and Torres Strait Islander men often nominate a “really rough” prison they want to go to, as it is perceived as a way of developing a hard masculinity and “becoming a real man”. One participant commented that many Aboriginal and Torres Strait Islander young men view being incarcerated as a “rite of passage”. This perception is deeply concerning for Elders and community leaders. As many participants highlighted, the aspiration to be incarcerated is additionally distressing given the high rates of physical and sexual assault that occurs in many Australian prisons.

### 3.6. Multiple Gaps in Sexual Assault Service Provision

Forum participants identified a number of critical gaps in sexual assault service provision pertaining to funding, resourcing, training and professional development. For instance, competitive tendering together with the time limited nature of funding often disrupts and damages current and future relationships between services working with Aboriginal and Torres Strait Islander communities. Participants suggested that this inadequate funding structure exacerbates the high turnover of workers which in turn negatively impacts service delivery for Aboriginal and Torres Strait Islander C&YP, their families and communities.

Participants agreed that there is a desperate need for more autonomous Aboriginal and Torres Strait Islander led services and community capacity building strategies to be funded as a way of empowering communities. Participants also emphasised the lack of resourcing and accessibility to services for C&YP who have sexually harmed in rural and remote communities, constitutes a significant service gap. Finally, participants noted that there is a lack of early intervention services geared to support C&YP who have been sexually assaulted and who are at risk of sexually harming others, especially between the ages 12–16 years.

## 4. Discussion

Literature exploring the association between child sexual abuse and family violence in the context of social and political oppression is limited [26]. However in a recent study investigating rates of domestic violence against women living in the occupied Palestinian territories, researchers concluded that “chronic exposure to institutionalised structural violence”, such “war, state repression, torture, and violent political conflicts” significantly increases the risk of gender-based, family violence and violence against children [32]. Although there are significant socio-political and historic differences, many parallels can be drawn between the experiences of Aboriginal and Torres Strait Islander people and other Indigenous peoples who have been oppressed by post-colonial structural violence [33]. Not only does ongoing colonial and post-colonial oppression create the context for interpersonal violence to occur, but it also creates innumerable barriers for victims of abuse to disclose violence and receive effective support.

Aboriginal and Torres Strait Islander C&YP and their families are likely to face what has been described as an “inescapable dilemma” ([7], p. 6), to maintain silence about abuse or risk involving what is perceived as racist and inequitable child protection and criminal justice systems—systems which have the power to break families and communities apart, exacerbating existing grief, loss and disconnection from kin and culture [7]. For these reasons disclosures of CSA are sometimes aggressively censured. Disclosures can also be silenced to avoid “shaming” Aboriginal identities and cultures as disclosures of abuse by an Aboriginal person is likely to be represented in the mainstream media as evidence of “Aboriginal dysfunction” and used to justify extreme interventions, such as child removal, rather than supportive, family and community led capacity building approaches [7,34].

For some Aboriginal and Torres Strait Islander and, indeed non-Aboriginal C&YP, making disclosures can be a process encumbered by community and peer perceptions that the young people are to blame for their own victimisation. Disclosures may also be inhibited in families and communities where sexuality, consent and sexual assault are considered to be taboo subjects [35]. Discourses of blame and shame along with active attempts to stifle disclosures leave C&YP vulnerable to ongoing sexual violence and exploitation, especially as C&YP often initially make disclosures to family and friends [35]. The double trauma of being blamed and shamed for being sexually assaulted adds another layer of alienation and distress for boys and young men, girls and young women. However as the *Yarn Up* participants identified, sexual assault and double trauma must be understood as highly gendered experiences. Thus sexual assault workers should not assume that sexual assault or healing is the same experience across gender identities. The “Yarning Together Without Shame” project held in Queensland (2004) was a sexual health workshop for young Aboriginal people and their parents to explore the “place of shame” around these issues [36]. The workshop had several positive outcomes including; “strengthening local capacity and facilitating the “natural networks” used by the young people to more effectively meet their sexual health needs” ([36], p. 15). This project highlights the potential for community led capacity building initiatives.

Forum participants identified that the Western-centric service delivery model is the most significant barrier for Aboriginal and Torres Strait Islander C&YP in accessing sexual assault services. This view echoes the findings of a research project conducted in the United States, in which women of colour reported that they feel reluctant to access support from sexual assault services that are “predominately directed and staffed by white staff members” as they feel “their needs and concerns will be overlooked and not addressed” both because of pervasive racist attitudes and differences in cultural and worldviews [37]. The unquestioned dominance of the Western worldview also risks perpetuating assimilation and acculturation stress not only for Aboriginal and Torres Strait Islander clients and their communities but also for staff. The unrelenting experience of acculturation stress also creates a context for lateral violence.

Mary Hagan argues that health services must acknowledge that “cultural diversity and a connection to one’s own culture is the key to recovery” [38]. This idea was strongly echoed by the *Yarn Up* forum participants who passionately argued that Aboriginal Worldviews may be healing and transformative not only for Aboriginal and Torres Strait Islander people but also for non- Aboriginal C&YP families and communities. The meaningful incorporation of Aboriginal worldviews is inherently strengths-based as it “positions the community in an active partnership…so that the process of deciding what to do is (and how to respond) is integral to the community development and the healing process” ([39], p. 154). This framework also reverses the pervasive deficit discourse, which blames Aboriginal culture for problems within their communities [40]. There are many emerging examples of the role of culture in healing both locally and internationally, although there are a limited number of program and service evaluations which have adopted this approach. A qualitative study exploring Canadian First Nation’s men’s experiences of fathering suggested that community programs using traditional cultural practices was a source of “strength” for the men and played a role in healing from intergenerational trauma [41].

*Yarn Up* participants also stressed that it imperative for practitioners working across Worldviews grasp the incommensurable and untranslatable differences between Aboriginal and Western paradigms [22]. In other words, traditional Aboriginal and Torres Strait Islander Worldviews are paradigmatically different to the Western Worldview. As there are hundreds of Aboriginal and Torres Strait Islander cultures it is not possible to articulate a single Aboriginal Worldview [22,38]. However, the concept of *interconnectedness* or *interrelatedness* appears to be significant to many Aboriginal and Torres Strait Islander traditional cultures. For example, interconnectedness or “Kanyini” in Anangu Pitjantjatjara and Yankunytjatjara language, encompasses four inter-related concepts; “Tjukurrpa” (spirituality and knowledge of creation or “Dreaming”), “Ngura” (connection to place and land), “Walytja” (connection to family and kinship) and Kurunpa (connection to spirit or soul) [42]. In contrast the Western Worldview is characterised by hierarchy and individualism rather than collectivism, monotheism rather than polytheism.

It is clear from forum discussions, that the *meaningful* integration of Aboriginal and Torres Strait Islander Worldviews; interconnectedness with land-kin-spirit-culture, are crucial for the development of culturally safe and accessible sexual assault services. How this may be achieved in the context of Australian sexual assault services is complex. Tensions between these divergent Worldviews play out frequently in the human services. For instance, within the Western model of health service provision, credibility and legitimacy are afforded to those who have obtained an appropriate level of formal education and to those who hold positions of power within a workplace context [22]. Conversely in many traditional Aboriginal cultures, “greater credence is given to perceptions of an individual’s trustworthiness, independent of their credentials or position” ([22], p. 13). Similarly the lack of coordination between services for young sexual assault victims and services for young people who sexually harm others does not reflect the diversity of Aboriginal and Torres Strait Islander family structures. This service divide can intensify the feelings of disempowerment and disconnection experienced by families especially where sibling sexual abuse has occurred or when a young person who is sexually harming others has also been a victim of CSA [21,43]. These ideas were reinforced by the *Yarn Up* participants who stressed the importance of victims and those who sexually harm others services to form greater alliances and, wherever possible, integrated practices.

## 5. Conclusions

Overwhelmingly, the national *Yarn Up* forum participants identified that an collaborative “victim” and “those who sexually harm” service model which prioritises cultural safety by meaningfully incorporating Aboriginal and Torres Strait Islander Worldviews would be best placed to provide support to Aboriginal and Torres Strait Islander C&YP. In order for this to happen, sexual assault services must transform their Western-centric bias. This is possible by firstly, becoming better informed of the socio-political context of violence, secondly, by “taking a stand” against racism and lateral violence and thirdly, by “reaching out to the local Aboriginal community” to develop strong understandings of their unique Worldviews, culture and family and community dynamics ([7], p. 10). Safety is the cornerstone of healing, and in practice, this means prioritising cultural safety alongside the physical safety of Aboriginal and Torres Strait Islander C&YP [6]. For Aboriginal and Torres Strait Islander C&YP healing and justice cannot occur until sexual assault services become culturally safe [44]. Cultural safety is not static or definitive, but is rather is a dynamic and flexible process. Cultural safety relies on services establishing meaningful, accountable and equitable long-term relationships with communities built on an understanding of their cultures and worldviews as well as their unique needs and strengths. One way a service can begin this process is by informally “yarning” with communities for twelve months prior to establishing a sexual assault service. Cultural safety also means engaging C&YP in safe places in their communities. Moving beyond the limited notion of cultural competency, cultural safety directs service providers to engage in a process of critical reflection. It also means developing a skilled Aboriginal and culturally safe non-Aboriginal sexual assault workforce. Increasing the pool of specialist Aboriginal and Torres Strait Islander sexual assault workers can be achieved through providing on the job training with supported pathways to higher education.

The *Yarn Up* participants identified, community led initiatives together with sexual assault services need to develop gender-specific programs and resources. As this is an emergent area of practice further research is needed to develop clearer understandings of how sexual assault services can increase their awareness of the gendered experiences of violence. It was beyond the scope of this research project to involve Aboriginal and Torres Strait Islander C&YP participants. However future research should explore the place of culture in the healing journeys of young Aboriginal and Torres Strait Islander people and their families. Future research may also describe and evaluate service models, which utilize Cultural Safety and Aboriginal and Torres Strait Islander Worldviews. However, any evaluation strategy must also strongly reflect Aboriginal and Torres Strait Islander knowledges and understandings.

Cultural Safety and Aboriginal and Torres Strait Worldviews pose a needed challenge to the current service model to increase access to effective support and reduce the inequitable power relationship between services and those who access them [45]. These recommendations also resonate with demands from Aboriginal and Torres Strait Islander Elders, community leaders and activists for self-determined community-led and controlled initiatives to received long-term committed funding to address the effects of both interpersonal and systemic, violence, trauma and dislocation [8]. Fundamentally, the Australian sexual assault service system needs to fully appreciate that “even though non-Aboriginal professionals may try to walk in our shoes, it’s still their own feet they are feeling” ([46], p. 31).
